# Cytokines in the Progression of Pancreatic **β**-Cell Dysfunction

**DOI:** 10.1155/2010/515136

**Published:** 2010-11-14

**Authors:** Chunjiong Wang, Youfei Guan, Jichun Yang

**Affiliations:** Department of Physiology and Pathophysiology, Peking University Diabetes Center, Peking University Health Science Center, Beijing 100191, China

## Abstract

The dysfunction of pancreatic *β*-cell and the reduction in *β*-cell mass are the decisive events in the progression of type 2 diabetes. There is increasing evidence that cytokines play important roles in the procedure of *β*-cell failure. Cytokines, such as IL-1*β*, IFN-*γ*, TNF-*α*, leptin, resistin, adiponectin, and visfatin, have been shown to diversely regulate pancreatic *β*-cell function. Recently, islet-derived cytokine PANcreatic DERived factor (PANDER or FAM3B) has also been demonstrated to be a regulator of islet *β*-cell function. The change in cytokine profile in islet and plasma is associated with pancreatic *β*-cell dysfunction and apoptosis. In this paper, we summarize and discuss the recent studies on the effects of certain important cytokines on pancreatic *β*-cell function. The imbalance in deleterious and protective cytokines plays pivotal roles in the development and progression of pancreatic *β*-cell dysfunction under insulin-resistant conditions.

## 1. Introduction

 Diabetes is a chronic disease that occurs when pancreatic islets fail to produce sufficient insulin and/or the sensitivity of glucose-metabolizing tissues to insulin decreases. Chronic hyperglycemia may lead to serious damage to many organs and cause the impairment of insulin production and action. 

 The mechanisms of islet *β* cell failure are different in the progression of type 1 and type 2 diabetes. In the progression of type 1 diabetes, pancreatic islet *β* cells are mainly destructed by autoimmune-mediated apoptosis, leading to the loss of insulin production. Inflammatory cytokines play crucial roles in this process [1]. In the progression of type 2 diabetes, the failure of *β*-cell function and *β*-cell mass reduction are predominantly associated with the increase in circulating cytokines and in free fatty acids (FFAs) and with persistent hyperglycemia [2]. Chronic exposure of *β* cell to these mediators induces excessive production of reactive oxygen species (ROS) and activation of caspases, which inhibit insulin secretion and promote apoptosis of pancreatic *β* cells [3]. 

 In the past decades, it had been well established that inflammatory cytokines including IL-1*β*, TNF-*α*, and IFN-*γ* play a critical role in the pathogenesis of type 1 diabetes. At the early stage of type 1 diabetes, some immune cells such as lymphocytes and macrophages infiltrate into the islets of pancreas and secrete inflammatory cytokines, resulting in high concentrations of cytokines within islets [1]. Chronic exposure of *β* cells to IL-1*β*, TNF-*α*, and IFN-*γ* finally induces islet dysfunction and *β* cell apoptosis. Since the discovery of leptin and other adipose-derived hormones, it has been realized that adipose is an endocrine organ besides being the main energy reservation tissue [4]. Adipocytokine is a general term of adipose-specific cytokines, such as leptin, resistin, adiponectin, visfatin, and omentin, and nonadipose-specific cytokines such as IL-6, IL-1*β*, and TNF-*α* [5, 6]. Cytokines including IL-1*β*, leptin, resistin, and adiponectin have been shown to play important roles in the development of pancreatic *β*-cell dysfunction and type 2 diabetes. Recently, PANcreatic DERived factor (PANDER, FAM3B), a cytokine-like protein, had been shown to be a regulator of pancreatic *β*-cell function [7–9].

 Depending on their roles in regulating pancreatic *β*-cell function, some cytokines are protective, others can be detrimental. For instance, chronic exposure of islets to some cytokines such as IL-1*β*, IFN-*γ*, TNF-*α*, and resistin inhibits insulin secretion and induces apoptosis of *β* cells. Other cytokines such as adiponectin and visfatin exert protective effects on pancreatic *β* cell function. In addition to circulating cytokines, islets also produce a variety of cytokines in response to physiologic and pathologic stimuli, and these locally produced cytokines play important roles in regulation of pancreatic *β*-cell function as well [10, 11]. To maintain the normal pancreatic *β*-cell function, the deleterious and protective cytokines need to be balanced. The abnormal change in cytokine profile in islet and plasma is associated with pancreatic *β*-cell dysfunction and type 2 diabetes [10, 12]. In this paper, recent findings regarding the effects of cytokines including IL-1*β*, IFN-*γ*, TNF-*α*, leptin, resistin, adiponectin, visfatin, and PANDER on pancreatic *β*-cell dysfunction and type 2 diabetes will be summarized and discussed.

## 2. IL-1*β*/IFN-*γ*/TNF-*α*

IL-1*β*-mediated pancreatic *β*-cell dysfunction and apoptosis are involved in the pathogenesis of pancreatic *β*-cell dysfunction and type 2 diabetes. Short-time pretreatment of pancreatic *β* cells with IL-1*β*, IFN-*γ*, and TNF-*α*, alone or in combination, results in significant inhibition of insulin secretion in the absence or presence of stimulatory glucose concentration [13, 14]. Chronic exposure of pancreatic *β* cell to IL-1*β* activates the expression of inducible nitric oxide synthase (iNOS) and results in excessive production of nitric oxide (NO), which interferes with electron transfer, inhibits ATP synthesis in mitochondria, and induces the expression of proinflammatory genes [15, 16]. A decrease in cellular ATP content inhibits insulin secretion and results in cell dysfunction. It has been widely accepted that Nuclear Transcription Factor-*κ*B (NF-*κ*B) predominantly mediates IL-1*β*- or other cytokine-induced activation of iNOS in pancreatic *β* cells [17–19]. Persistent activation of NF-*κ*B induces a sustained decrease in expression of *β*-cell-specific proteins including insulin, GLUT-2, and PDX-1 concomitant with an increase in iNOS expression [20]. Sulforaphane, radix clematidis, guggulsterone, or other molecules has been shown to protect pancreatic *β* cell from apoptosis induced by cytokines including IL-1*β* and IFN-*γ* via inhibition of NF-*κ*B activation and iNOS expression [21–23]. Overexpression of MnSOD also protects *β* cells from IL-1*β* or other cytokine-induced apoptosis by repressing NF-*κ*B activation and iNOS expression [24]. NF-*κ*B1 (p50)-deficient mice are not susceptible to multiple low-dose streptozotocin-induced diabetes [25]. In contrast to persistent activation, transient activation of NF-*κ*B may be beneficial to insulin secretion from pancreatic islets at the early stage of cytokine stress [20, 26]. However, cytokines including IL-1*β*, IFN-*γ*, and TNF-*α* have also been reported to inhibit insulin secretion and induce apoptosis of *β* cell via iNOS-independent pathway [27, 28]. Endoplasmic reticulum (ER) stress-mediated apoptosis has been proposed as an additional important mechanism for IL-1*β*-mediated pancreatic *β* cell death. Pretreatment of *β* cells (primary islet *β* cells and MIN6 cells) with 4-Phenyl butyric acid (PBA) to alleviate ER stress significantly reduces IL-1*β*-induced cell apoptosis [29]. PBA may partially alleviate IL-1*β*'s deleterious effect on *β* cell by depleting ER Ca^2+^ and activating c-Jun NH(2)-terminal kinase (JNK) signaling pathway [29]. IL-1*β* and IFN-*γ* in combination markedly decrease the sarcoendoplasmic reticulum pump Ca^2+^ ATPase 2b (SERCA2b) protein expression and deplete ER Ca^2+^ stores by stimulating NO synthesis, which subsequently activates the ER stress pathway [30]. IL-1*β* plus IFN-*γ* also upregulates the BH3-only protein, DP5, which induces ER stress and consequently triggers *β* cell apoptosis [31]. Maedler and colleagues reported in 2002 that incubation of human islets with high concentration of glucose (33.3 mM) for 20 hours significantly induced IL-1*β* production and locally produced IL-1*β* exerted deleterious effects on human islet function [32]. This suggests that islet-produced IL-1*β* maybe be involved in glucotoxicity on islet *β* cell. In support, IL-1*β* expression is shown to be increased in islets from type 2 diabetic patients [33]. However, Welsh and colleague report that stimulation with 11 and 28 mM glucose for 48 hours or 7 days fails to affect the expression of IL-1 receptor antagonist (IL-1ra), Fas, IkB-*α*, or monocyte chemoattractant protein (MCP-1) in human islets. The authors further show that high glucose fails to induce IL-1*β* production in human islets [34]. Overall, although whether glucose regulates the expression of IL-1*β* or IL-1ra in human islets remains controversial [34], it has been well established that local and/or systemic IL-1*β*'s play an important role in the progression of islet dysfunction and *β* cell apoptosis in type 2 diabetes. Adenoviral-mediated overexpression of IL-1ra increases *β*-cell replication in rat pancreatic islets [35]. IL-1Ra treatment ameliorates hyperglycemia of high-fat-diet- (HFD-) induced mice. In vitro, IL-1ra protects islets of HFD-treated mice from *β*-cell apoptosis, induces *β*-cell proliferation, and improves glucose-stimulated insulin secretion [36]. Consistently, administration of IL-1*β*-neutralizing antibody for 13 weeks significantly reduces glycated hemoglobin (0.45%), serum proinsulin (2.1 ± 0.2 versus 4.8 ± 0.9 ng/mL), and insulin levels (3.6 ± 0.5 versus 5.2 ± 1.4 ng/mL), and improves islet function in HFD-induced diabetic mice [37]. Treatment of diabetic GK rats with IL-1ra attenuates hyperglycemia, reduces the proinsulin/insulin ratio, and improves insulin sensitivity. In addition, the expression of islet-derived proinflammatory cytokines including IL-1*β* and TNF-*α* is reduced by IL-1ra treatment with amelioration of islet inflammation [38, 39]. IL-1ra also protects human islets from IL-1*β*-induced production of NO, impairment in glucose-stimulated insulin secretion, and apoptosis of *β* cells [15, 40]. Pioglitazone also protects human islet *β* cells from IL-1*β*-induced apoptosis by blocking NF-*κ*B activation [41]. Patients with type 2 diabetes receiving subcutaneously a daily dose of 100 mg of anakinra, a recombinant human IL-1ra, for 13 weeks show a significant decrease in glycated hemoglobin level and fasting blood glucose, ratio of serum proinsulin to insulin, and IL-6 and C-reactive protein levels while show an increase in serum C-peptide level [42, 43]. Clearly, these studies in animal models and human or human islets strongly suggest that blockade of IL-1*β* signaling pathway will improve *β*-cell dysfunction and ameliorate hyperglycemia. 

It has been previously reported that the use of TNF-*α* or IFN-*γ* alone fails to induce *β* cell apoptosis, whereas in combination they markedly induce *β* cell death. Interferon regulatory factor 1 (IRF-1) may mediate IFN-*γ*/TNF-*α*-induced apoptosis of pancreatic *β* cells. IFN-*γ* induces the expression of IRF-1, which makes insulinoma cells susceptible to TNF-*α* [44]. X-linked inhibitor of apoptosis protein (XIAP), an antiapoptotic protein, can protect pancreatic *β* cells from being damaged by IFN-*γ*/TNF-*α* toxicity. Overexpression of XIAP abrogates TNF-*α* induced apoptosis of insulin-secreting MIN6N8 cells via inhibition of caspase activation, whereas downregulation of XIAP augments MIN6N8 cell apoptosis induced by TNF-*α* and IFN-*γ* [45]. Moreover, the amplitude of high-voltage-activated Ca^2+^ currents has been demonstrated to be increased in MIN6N8 insulinoma cells exposed to IFN-*γ* and TNF-*α*, resulting in an increase in cytosolic Ca^2+^ concentration and activation of calpain and calcineurin. Activated calcineurin mediates dephosphorylation of the proapoptotic protein BAD. Intracellular events such as mitochondrial dysfunction and caspase activation are also involved in apoptosis of pancreatic *β* cell involving Ca^2+^ channel activation [46]. In addition, excessive production of ROS, decrease in mitochondrial transmembrane potential, activation of JNK/SAPK and P53 pathways, upregulation of suppressor of cytokine signaling proteins (SOCS), and activation of NF-*κ*B and iNOS may also be involved in the underlying mechanisms accounting for IFN-*γ*/TNF-*α*-induced apoptosis of pancreatic *β* cells [47–49]. In vivo, the decrease in circulating TNF-*α* level has been reported to be involved in improvement of *β*-cell function of type 2 diabetic patients receiving transient intensive insulin therapy [50].

## 3. Leptin

Since the discovery of adipocyte-derived hormone leptin in 1994 [4], several other adipocyte-derived cytokines have been identified. Leptin is one of the most important cytokines secreted by adipose, and it plays vital roles in controlling food intake and body energy balance [51]. Leptin- or leptin-receptor-deficient mice exhibit severe obesity and diabetes [52]. Recently, leptin has been shown to directly regulate insulin secretory process from pancreatic islets. Leptin receptors have been shown to be expressed in rat islets and murine-derived *β*-TC3 cells [53]. Covey and colleagues report that mice with deletion of leptin receptor in pancreatic *β* cells and hypothalamus develop obesity, fasting hyperinsulinemia, impaired glucose-stimulated insulin release, and glucose intolerance [54]. Morioka and colleagues further confirm that mice with specific disruption of leptin receptor in pancreatic *β* cells develop more severe glucose intolerance when fed a high-fat diet due to impaired insulin secretion from *β* cells [55]. Consistently, we previously demonstrated that electroporational transfer of naked plasmid with human leptin gene into skeletal muscle of normal C57/B6 mice leads to increased circulating leptin level and decreased serum proinsulin level and fasting blood glucose [56]. Recently, Chen and colleagues have reported that overt type 2 diabetes in Akt1(+/−)Akt2(−/−) mice is due to markedly decreased leptin level and *β*-cell dysfunction. Hyperglycemia of Akt1(+/−)Akt2(−/−) mice is significantly attenuated by restoring plasma leptin level concomitant with increased circulating insulin level [57]. Leptin has also been reported to prevent pancreatic *β* cells from inducible apoptosis, and this may partially account for islet hypertrophy in obese rodents and patients. Leptin may exert its antiapoptotic effects on pancreatic *β* cells by reducing triglyceride accumulation, inhibiting NO production, increasing antiapoptotic protein Bcl-2, and reducing apoptotic protein Bax [58–60]. Recent findings indicate that IRS2-PI3K-Akt signaling axis plays a crucial role in *β*-cell proliferation [61, 62]. Leptin suppresses PTEN activity via CK2- (cyclin-dependent kinase-) dependent pathways and results in an increase in PIP3 availability, which activates PI3K/Akt signaling pathway in pancreatic *β* cells [63]. All these results suggest that leptin has a protective role on pancreatic *β* cells function. However, leptin has also been shown to inhibit insulin secretion of *β* cells via activation of ATP-regulated potassium (K_ATP_) channels, reduction in cellular cAMP level, and activation of PI3K-dependent activation of cyclic nucleotide phosphodiesterase 3B (PDE3B) signaling pathway [64, 65]. Supportively, Laubner and colleague show that leptin inhibits insulin biosynthesis in pancreatic *β* cells by activating suppressor of cytokine signaling 3 (SOCS3) [66, 67]. Leptin also suppresses acetylcholine-induced insulin secretion in isolated perfused chicken pancreas [68] and induces the expression of inflammatory genes in RINm5F insulinoma cells [69]. A recent study further indicates that mice with disrupted leptin signaling in *β* cells display hyperinsulinemia, insulin resistance, glucose intolerance, obesity, and reduced fasting blood glucose. The authors further propose that insulin resistance of these mice is due to excessive insulin secretion from pancreatic *β* cells [70]. Central fusion of leptin directly decreases insulin secretion capacity of pancreatic islets in rat model [71]. In contrast to the controversial observations in rodent models, leptin is likely to exert deleterious impact on human islet function. A clinical study reveals that in obese women after standardized weight reduction, improved pancreatic *β*-cell function is independently associated with the decreased leptin and increased adiponectin levels in circulation [72]. In vitro, leptin decreases the expression of IL-1ra and stimulates the release of IL-1*β* in human islets [73]. Another study from the same group further indicates that leptin impairs insulin secretion and induces apoptosis of *β* cells in the presence of 20 mM glucose via activation of c-JNK in human islets [74]. Leptin also impairs insulin secretion of human islets via inhibition of UPC2 expression or increase in potassium channel permeability [75, 76]. 

Overall, leptin is likely to exert diverse effects in regulation of pancreatic *β* cell function, and further research is still required to clarify its distinct role in various conditions.

## 4. Resistin

Resistin is another adipose-derived cytokine first described in 2001 [77]. Unlike the expression of resistin in mouse, human resistin is expressed primarily in macrophages but not in adipose [78]. Increased serum resistin level is associated with insulin resistance in rodents and human. It has been demonstrated that resistin impairs glucose tolerance and antagonizes insulin action, indicating that resistin may be an important cytokine linking obesity to diabetes [77]. Treatment of diabetic mice with resistin-neutralizing antibodies significantly ameliorates hyperglycemia of HFD-fed mice [77]. Resistin increases *β*-cell viability at physiological concentrations (10–20 ng/mL) [79]. In contrast, pretreatment of pancreatic *β* cells (*β* TC-6 or BRIN-BD11 cells) with pathological concentration of resistin (40 ng/mL) for 24 hours significantly reduces insulin receptor expression [79]. Resistin induces apoptosis of rat insulinoma cell RINm5F at the concentration of 200 ng/mL via activation of caspase-3 and NF-*κ*B, which can be blocked by TIMP-1, an inhibitor of Metalloproteinase-1 [80]. When the plasma concentration of resistin is elevated by adenoviral-mediated delivery of resistin, mice exhibit impaired insulin secretion in response to glucose. In vitro, pretreatment of pancreatic islets with resistin augments insulin release at basal glucose concentration (2.8 mM) whereas inhibits insulin release at stimulatory glucose concentration (8.3 mM). The authors further show that resistin impairs insulin secretion of islets by inducing SOCS3 expression and inhibiting Akt phosphorylation [81]. Resistin is also expressed in human islets, and its expression is upregulated in insulin-resistant status, suggesting that islet-produced resistin may be involved in the progression of *β*-cell dysfunction in insulin-resistant condition [82]. Overall, increased circulating resistin level in obese or insulin-resistant status impairs insulin secretion from islets, resulting in deterioration of glucose homeostasis. Targeting resistin may represent a novel therapeutic strategy for islet dysfunction and type 2 diabetes.

## 5. Adiponectin

Adiponectin, also known as Acrp-30 (adipocyte complement-related protein of 30 kDa), apM1 (adipose most abundant gene transcript1), AdipoQ, and GBP28 (gelatin-binding protein of 28 kDa), is another adipose-derived cytokine. It is one of the most abundant circulating proteins with a concentration greater than 5 mg/mL [83]. Adiponectin protein is composed of an N-terminal collagenous domain and a C-terminal globular domain. In vivo, adiponectin exists in full-length form or as globular fragment. Adiponectin in plasma is in its full-length form [84]. Adiponectin exerts its physiological functions by binding to two subtypes of adiponectin receptors, which are designated as AdipoR1 and AdipoR2, respectively. AdipoR1 is abundantly expressed in muscle, and AdipoR2 is mainly expressed in liver. The demonstrated physiological functions of adiponectin include insulin-sensitizing, antiatherogenic, and anti-inflammatory effects [85]. Recently, there has been an increasing evidence that adiponectin is also involved in the regulation of pancreatic *β*-cell function. Plasma adiponectin level is significantly lower in type 2 diabetic patients when compared with control subjects [86]. Lower plasma adiponectin level is associated with pancreatic *β*-cell dysfunction in women during pregnancy [87]. In Hispanic women with recent gestational diabetes mellitus, a decline in *β*-cell compensation for insulin resistance is associated with decreased circulating adiponectin level [88]. Supportively, low plasma adiponectin level has been reported to predict abnormal pancreatic *β*-cell function in Chinese men [89]. Both AdipoR1 and AdipoR2 have been shown to be functionally expressed in human and rat pancreatic *β* cells, and their expression can be upregulated by oleate (unsaturated fatty acid) but not palmitate (saturated fatty acid) [90]. A recent study shows that the expression of AdipoR2 in murine-derived *β*-cell line NIT-1 cells is increased under acute hyperlipidemic stress. In contrast, chronic hyperlipidemic stress significantly downregulates AdipoR2 in NIT-1 cells, which can be reversed by activation of peroxisome proliferator-activator receptor *α* (PPAR*α*) [91]. The expression of AdipoR1 is decreased, whereas the expression of AdipoR2 remains unchanged in the islets of ob/ob mice [92]. Pretreatment with adiponectin prevents INS-1 cells from insulin secretion dysfunction and apoptosis induced by inflammatory cytokines, free fatty acids, and high glucose [93, 94]. Adiponectin augments insulin secretion in islets from normal rat or HFD-treated mice at high glucose concentrations [95, 96]. Brown and colleagues report that globular adiponectin stimulates PDX-1 expression by 450% and decreases LPL (lipoprotein lipase) expression by 45% in rat *β*-cell line BRIN-BD11 cells [97]. In human islets, pretreatment with full-length adiponectin induces phosphorylation of acetyl coenzyme A carboxylase (ACC) without significant impact on basal or glucose-stimulated insulin secretion [98]. This suggests that adiponectin may repress the synthesis of fatty acids and prevent lipid deposition in human *β* cells. In the same study, the authors also report that adiponectin fails to prevent human islet cells from apoptosis induced by FFAs [98]. Overall, adiponectin is a positive regulator of pancreatic *β* function and may be a putative target for treatment of islet dysfunction and type 2 diabetes.

## 6. Visfatin

In 2005, visfatin mRNA was identified from the visceral fat using differential display PCR technology [99]. Visfatin had been previously identified as a pre-B-cell colony-enhancing factor (PBEF) or nicotinamide phosphoribosyltransferase (Nampt), a 52-kd cytokine expressed in lymphocytes. When compared with wild-type mice (visfatin^+/+^), heterozygous visfatin^+/−^mice show lower circulating visfatin level and higher plasma glucose concentration [99]. In vitro, visfatin enhances glucose uptake in 3T3-L1 preadipocytes and suppresses gluconeogenesis in H4IIEC3 hepatocytes. The authors further show that visfatin can mimic the effects of insulin by binding to the insulin receptor and activating insulin signaling pathway [99]. Supportively, visfatin regulates glucose uptake, cell proliferation, and type I collagen production in human osteoblasts in an insulin-like manner [100]. Serum visfatin level is elevated in patients with type 2 diabetes and decreased after intensive glycemic control [101]. It is also reported that visfatin synthesis is increased in adipose tissue under diabetic status, leading to activation of NF-*κ*B and systemic inflammation [102]. Revollo and colleagues report that Nampt (visfatin) heterozygous (Nampt+/−) mice show impaired glucose tolerance and reduced glucose-stimulated insulin secretion, suggesting that visfatin may be involved in regulation of insulin secretion in *β* cells. Controversially, the authors fail to observe its insulin-mimetic effects [103, 104] such as inducing preadipocyte differentiation, activation of insulin receptor and Akt, as previously reported [99]. Furthermore, visfatin also fails to stimulate glucose transport and mimic the lipolysis inhibition effect of insulin in human adipocyte [105]. Brown and colleagues report that visfatin upregulates the mRNA expression of insulin (9-fold to control) and enhances insulin secretion by 46% in murine-derived *β*-TC6 cells at low glucose. These effects of visfatin can be blocked by FK866, a specific inhibitor of Nampt [106]. Overall, visfatin may have protective effect on pancreatic *β*-cell function, but further research is required to clarify its distinct roles.

## 7. PANcreatic DERived Factor (PANDER, FAM3B)

PANDER (PANcreatic DERived factor, FAM3B) is a novel cytokine that has been recently cloned and identified using an algorithm, ostensible recognition of folds (ORF), searching for novel cytokines based on their predicted secondary structure [11]. The rationale for this approach is that the secondary structure of cytokines is highly conserved through evolution. Many cytokines are four-helix bundles with disulfide bridges. This approach has allowed the identification of a novel cytokine family consisting of 4 members: 2–19 (FAM3A), EF-7 (FAM3D), FAM3C, and FAM3B in 2002. 

Northern blot and immunohistochemical assays reveal that FAM3B is highly expressed in pancreatic islets [11]. FAM3B is thus also called PANcreatic DERived factor (PANDER) in subsequent studies [7]. PANDER is a 235-amino acid protein with a secretion signal peptide [11]. In vitro, we have demonstrated that recombinant PANDER pretreatment or viral-mediated overexpression of PANDER inhibits insulin secretion and induces pancreatic *β*-cell apoptosis of rodent and human islets [7, 8, 107]. IFN-*γ* has been shown to regulate PANDER expression in mouse islets in a dose- and time-dependent manner, suggesting that PANDER may be involved in IFN-*γ*-mediated apoptosis of islet *β* cells [108]. Furthermore, glucose potently activates the PANDER promoter activity in primary pancreatic islets and *β*-cell lines [101, 109]. More recently, Burkhardt and colleagues report that the PANDER promoter activity is also regulated by PDX-1, confirming that the expression of PANDER is regulated in a manner similar to insulin in islets [110]. These observations have suggested that PANDER may have a potential role in the regulation of *β*-cell function and glucose homeostasis as a locally produced cytokine in islets.

 In addition, PANDER protein is cosecreted with insulin from pancreatic *β* cells through a similar Ca^2+^-dependent regulatory mechanisms [9], suggesting that PANDER may also act on other tissues or cells as an endocrine factor. To identify the target tissues of PANDER, the in vitro ^125^I-PANDER saturation and competitive binding assays have been performed using tissue membranes. The binding results indicate that PANDER specifically binds to the liver cell membrane. Cross-linking experiments further confirm that PANDER interacts with some unknown protein on the liver membrane. In contrast, PANDER does not bind to the membrane of pancreas, muscle, kidney, and heart [111]. In HepG2 cells, PANDER pretreatment significantly inhibits insulin-stimulated activation of IR, IRS-1, PI3K, and Akt. These observations have suggested that liver is a novel target for islet-secreted PANDER. In addition, PANDER fails to induce cell apoptosis of HepG2 cells. The physiological role of PANDER is to some extent similar to that of amylin, also known as islet amyloid polypeptide (IAPP). Amylin is another islet-specific protein which is cosecreted with insulin from *β* cells [112]. Normally, amylin inhibits glucagon secretion, delays gastric emptying, and inhibits food intake [113]. However, chronic hyperglycemia stimulates amylin production in insulin-resistant condition. Excessive amylin will be deposited as amyloid in islets and will result in islet dysfunction [114, 115]. Interestingly, PANDER-deficient mice show glucose intolerance due to impaired insulin secretion from pancreatic *β* cells [116]. Because the expression and secretion of PANDER is similarly regulated by glucose as insulin, it is reasonable to speculate that PANDER may regulate insulin secretion process [9, 109, 110, 116, 117]. In addition, pancreatic *α* cells also secrete PANDER in response to L-arginine and insulin [118]. 

Overall, chronic hyperglycemia and compensatory increase in insulin secretion ability of pancreatic *β* cells and islet hypertrophy may result in increased production of PANDER in islets. Excessive PANDER may negatively regulate islet function as a local cytokine and deteriorate hepatic glucose metabolism as an endocrine factor. Clearly, PANDER may be a novel linker between insulin resistance (prediabetes) and type 2 diabetes.

## 8. Summary and Perspective

It is likely that crosstalk among cytokines in islets or other tissues may also be widely involved in regulation of pancreatic *β*-cell function. For example, TNF-*α* neutralization increases circulating adiponectin level and decreases resistin level in patients with the metabolic syndrome [119]. Leptin represses resistin expression in adipose [120]. Leptin also decreases IL-1ra expression and increases IL-1*β* release in islets [73, 120]. IFN-*γ* upregulates PANDER expression in *β* cells [108]. Insulin regulates the expression and secretion of various cytokines in islets and adipose, which may in return regulate insulin secretion from islets [118, 121–123]. 

**Figure 1:**
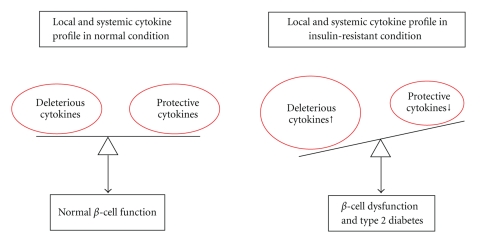
Cytokines play important roles in regulation of pancreatic *β*-cell function. The disturbed balance of deleterious and protective cytokines in islets and plasma plays crucial roles in the development and progression of *β*-cell dysfunction and type 2 diabetes.

Overall, cytokines are widely involved in the regulation of pancreatic *β*-cell function. In insulin-resistant status, the levels of deleterious cytokine in islet *β* cells and plasma increase, whereas the levels of protective cytokines decrease. This abnormal change in local and circulating cytokines plays an important role in triggering *β*-cell dysfunction and type 2 diabetes (Figure 1). Restoring the normal cytokine profile in *β* cells and plasma may hold great promise for treatment of *β*-cell dysfunction and type 2 diabetes.
